# Local Application of Transcutaneous Carbon Dioxide Paste Decreases Inflammation and Accelerates Wound Healing

**DOI:** 10.7759/cureus.19518

**Published:** 2021-11-12

**Authors:** Rika Amano-Iga, Takumi Hasegawa, Daisuke Takeda, Aki Murakami, Nanae Yatagai, Izumi Saito, Satomi Arimoto, Yasumasa Kakei, Akiko Sakakibara, Masaya Akashi

**Affiliations:** 1 Oral and Maxillofacial Surgery, Kobe University Hospital, Kobe, JPN

**Keywords:** transcutaneous co2, hypoxia, blood flow, angiogenesis, wound healing

## Abstract

Introduction: Delayed wound healing after surgery lowers the long-term quality of a patient’s life and leads to discomfort and pain. However, treatments for wound healing are often difficult and have not yet been fully established. In this study, we investigated the effect of a special paste that can be administered transdermally and holds a non-gaseous carbon dioxide (CO_2_) source in its carrier, which can be applied to the head and neck region for wound healing in a rat skin defect model.

Methods: Forty-eight Sprague Dawley rats were randomized into control and CO_2_ groups. We punched a 6.2-mm wound on the back of each rat. The control rats were left untreated, whereas rats in the CO_2_ group were treated with the CO_2_ paste every day after surgery. We evaluated wound healing 3, 7, 14, and 21 days after wounding by analyzing the diameter of the wound, gene expression of inflammatory markers vascular endothelial growth factor (VEGF), transforming growth factor (TGF)-β, hypoxia-inducible factor (HIF)-1α, interleukin (IL)-1β, and IL-6 using quantitative real-time polymerase chain reaction, hematoxylin and eosin, and immunohistochemical staining patterns.

Results: Rats in the CO_2_ group showed accelerated wound healing compared to those in the control group. Furthermore, VEGF and TGF-β were overexpressed, whereas HIF-1α, IL-1β, and IL-6 were downregulated in the rats treated with CO_2_. Immunohistochemical analysis also revealed similar patterns of expression.

Conclusion: Taken together, the CO_2_ paste promoted wound healing by regulating the hypoxic environment, reducing inflammation, and accelerating angiogenesis.

## Introduction

The skin functions as a protective physical barrier against a variety of environmental insults and is critical for homeostasis [1]. Wound dressings are used for the temporary treatment of damaged and wounded skin [2]. In oral surgery, the skin protects against possible infection after head and neck surgery and facial injuries. Delayed wound healing after surgery or injury causes discomfort and pain, thereby lowering the long-term quality of life in patients. Thus, rapid wound healing is imperative.

After head and neck surgery and injury, a delay in wound healing is likely to cause infection, prolonged and expensive hospital stay, and further delay in additional treatment [3]. Microwave radiation and lasers have been used to enhance wound healing. However, these treatment strategies are associated with disadvantages, such as negative effects on the heart and high costs [4]. Transcutaneous carbon dioxide (CO_2_) therapy has been developed for use in humans and animal models to evaluate its efficacy in treating various conditions, for example, medical and beauty treatments [5-7]. This therapeutic strategy is safe and benefits human health by affecting various biological processes. We have previously reported the use of a topical cutaneous CO_2_/hydrogel to accelerate the repair of a fracture and enhance angiogenesis and blood ﬂow [8]. Transcutaneous CO_2_ therapy also improves the blood flow and angiogenesis in skin flaps [9] and stimulates muscle injury repair [10].

However, studies done so far have used gaseous CO_2_ that cannot be applied to the head and neck region. A CO_2_ paste has been developed that reacts with the moisture on the surface of the skin without letting pure carbon dioxide gas generate carbon dioxide in the applied material and allowing efficient absorption of carbon dioxide from the skin. Thus, this study aims at using CO_2_ paste to repair skin defects in Sprague Dawley rats and investigating wound healing using gene expression analysis and immunohistochemistry.

## Materials and methods

Animals

Healthy, adult male seven-week-old Sprague Dawley rats were procured from Charles River Laboratories Inc (Tokyo, Japan). Six rats each were randomly divided into two groups: the CO_2_-treated group (CO_2_ group: n = 6 rats/time point) and control group (n = 6 rats/time point). All animal experiments were performed according to the Kobe University Animal Experimentation Regulations (approval number P171203). All rats used in the experiment did not receive any other medication. The rats were analyzed 3, 7, 14, and 21 days after wound generation.

Wound generation

Before wounding, the rats were anesthetized using isoflurane (Pfizer Inc., NY, USA) in O_2_ and injected intraperitoneally with 45 mg/kg body weight of pentobarbital (Kyoritsu Seiyaku, Tokyo, Japan). The dorsal skin was shaved following which a 6.2-mm wound was punched on the back of each rat.

CO_2_ paste treatment

The CO_2_ paste (comprising sodium hydrogen carbonate and malic acid that generated CO_2_ gas) was obtained from CO_2_TECH (Kobe, Japan). The CO_2_ paste contained 1,3-butylene glycol (about 80%), and other components (about 20 %) including sodium hydrogen carbonate, malic acid, sodium dihydrogen phosphate, alkyl-modified carboxyvinyl polymer, and carboxyvinyl polymer. The wound on the rats in the CO_2_ group was covered with the CO_2_ paste for 10 minutes every day after surgery. The control group rats were wounded and the wounds were left untreated without the CO_2_ paste.

Analyzing wound closure

The wounds were photographed 0, 3, 7, 14, and 21 days post-wounding and were measured using ImageJ (a public domain software available at https://imagej.nih.gov/ij/). Wound closure was calculated using the following formula: Wound closure (%) = [(wound area on day 0 - wound area on the indicated day)/wound area on day 0] × 100.

Quantitative real-time polymerase chain reaction (PCR)

We measured the mRNA levels of vascular endothelial growth factor (VEGF), hypoxia-inducible factor-1α (HIF-1α), transforming growth factor-β (TGF-β), interleukin-1β (IL-1β), and interleukin-6 (IL-6). The rats were sacrificed 3, 7, 14, and 21 days after injury. Tissue samples were collected from around the wound from which total RNA was extracted using TRIzol (Invitrogen, Carlsbad, CA) and treated using the RNeasy Mini Kit (Qiagen, Valencia, CA). cDNA was synthesized (1,000 ng of total RNA) using the High-Capacity cDNA (complementary DNA) Reverse Transcription Kit (Applied Biosystems, Foster City, CA). mRNA levels were quantified using the Step One Real-Time PCR System (Applied Biosystems, Foster City, CA). Real-time PCR (20 µl) was performed using 0.5 μM forward primer, 0.5 μM reverse primer, 1 µl of template cDNA, and 10 µl (2×) Power SYBR Green Master Mix (Applied Biosystems, Foster City, CA). The PCR conditions were as follows: 95°C for 10 min followed by 40 cycles at 95°C for 15 s and 60°C for 1 min. Target gene expression was normalized to β-actin and fold-change was calculated using the 2-ΔΔCT method (Applied Biosystems, Foster City, CA). β-actin, VEGF, HIF-1α, and IL-6 primers were obtained from Invitrogen and those for TGF-β and IL-1β were procured from Qiagen. The primer information is presented in Tables 1-2.

**Table 1:** Specific primer sequence for real-time polymerase chain reaction analysis VEGF = Vascular endothelial growth factor; HIF-1α = Hypoxia-inducible factor 1α; IL-6 = Interleukin-6; Fw = Forward primer; Rv = Reverse primer; A = Adenine; G = Guanine; C = Cytosine; T = Thymine

Gene name	Primer sequence (5’-3’)
β-actin	Fw: GAT GAG ATT GGC ATG GCT TT Rv: CAC CTT CAC CGT TCC AGT TT
VEGF	Fw: GTA TAT CTT CAA GCC GTC CTG TGT G Rv: GAT CCG CAT GAT CTG CAT AGT GAC
HIF-1α	Fw: CCT GCA CTG AAT CAA GAG GTT GC Rv: CCA TCA GAA GGA CTT GCT GGC T
IL-6	Fw: GGT CTT CTG GAG TTC CGT TTC Rv: GGT CTT GGT CCT TAG CCA CTC

**Table 2:** Primers used for real-time polymerase chain reaction analysis TGF-β = transforming growth factor β; IL-1β = Interleukin-1β

Gene name	Primer						
TGF-β	Rn_Tgfb1_1_SG QuantiTect Primer Assay cat no: QT00187796						
IL-1β	Rn_Il1b_1_SG QuantiTect Primer Assay cat no: QT00181657						

Hematoxylin and eosin (H&E) staining

The tissues were fixed in 4% paraformaldehyde and embedded in paraffin wax. The paraffin-embedded 6-µm thick samples were sectioned using a microtome and stained with H&E. We defined wound healing as a condition indistinguishable from tissues adjacent to the site of the wound. Images of the sections were captured using BZ-X700 (Keyence, Osaka, Japan) at a magnification of ×200.

Immunohistochemistry

Immunohistochemistry was performed using the tissue sections from the wound to detect the protein levels of VEGF, HIF-1α, and TGF-β (10 each). The formalin-fixed and paraffin-embedded skin tissues were pretreated with proteinase K (Dako company, CA, USA), quenched with 0.05% H_2_O_2_, and incubated overnight at 4℃ with the following primary antibodies in Can Get Signal lmmunostain Solution A (Toyobo, Osaka, Japan): mouse anti-VEGFA antibody (1:200, Abcam, Cambridge, UK) and anti-HIF-1α antibody (1:100, Abcam). The rabbit anti-TGFB1 antibody (1:100, Abcam) was heat-treated with ethylenediaminetetraacetic acid (EDTA) instead of proteinase K. Following washes using phosphate-buffered saline, we incubated the sections with peroxidase-conjugated labeled anti-mouse (cat no. 424131) or anti-rabbit (cat no. 424141) antibodies (Histofine Simplestain MAX-PO; Nichirei, Tokyo, Japan) for 30 min. Following staining with diaminobenzidine and counterstaining with hematoxylin, the tissue sections were visualized and photographed using the BZ-X700 fluorescence microscope (Keyence, Osaka, Japan) at a magnification of ×400. The positively stained areas were quantified using the Hybrid cell count BZ-H3C software (Keyence, Osaka, Japan).

Statistical analysis

Data collection and statistical analyses were performed using Excel-Toukei 2012 (Social Survey Research Information Co. Ltd., Tokyo, Japan). Data have been represented as mean ± standard error. Data from the two groups were analyzed using the Mann-Whitney U-test. A value of p <0.05 was considered statistically significant.

## Results

Rate of wound healing

**Figure 1:**
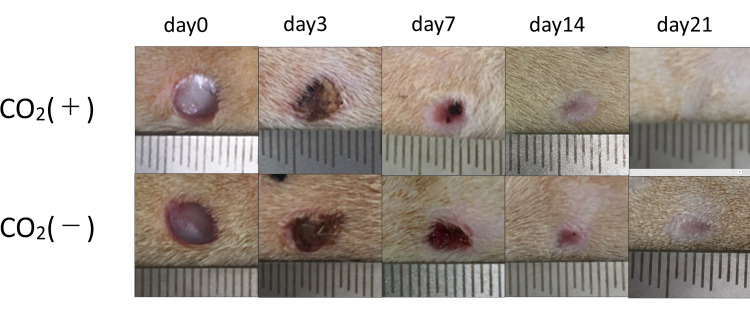
Representative photographs of the wound on days 0, 3, 7, 14, and 21.

Figure 1 shows the representative photographs of the wound on days 0, 3, 7, 14, and 21.

**Figure 2:**
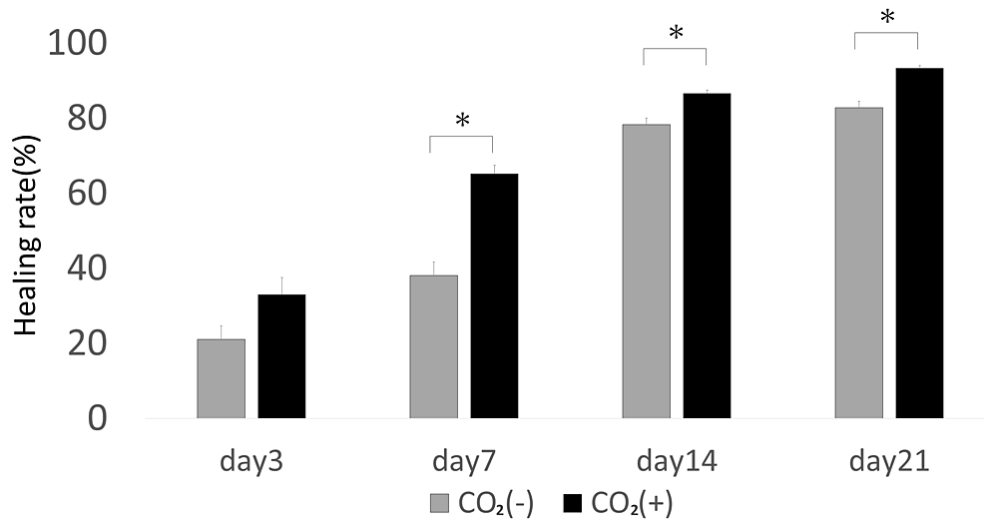
Wound healing rate (percentage) on days 3, 7, 14, and 21 * = p＜0.05

Figure 2 shows the percentage of wound healing in the control and CO_2_ groups. Three days after the wounds were punched, the rates of wound healing in the control and CO_2_ groups were 21.04% ± 3.62% and 32.98% ± 4.48%, respectively. On day 7, the rates of wound healing in the control and CO_2_ groups were 38.02% ± 3.66% and 65.11% ± 2.33%, respectively (p < 0.05). On day 14, the rates of wound healing in the control and CO_2_ were 78.24% ± 1.75% and 86.57% ± 0.90%, respectively (p < 0.05). On day 21, the rates of wound healing in the control and CO_2_ groups were 82.74% ± 1.75% and 93.30% ± 0.68%, respectively (p < 0.05). Thus, the rates of wound healing were significantly higher after three days in rats treated with the CO_2_ paste as compared to those in the control rats. 

Gene expression profiles

**Figure 3:**
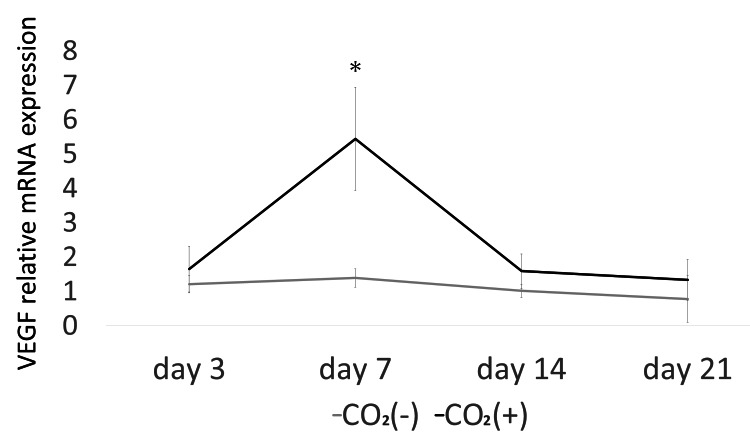
The mRNA expression of VEGF. The mRNA expression was evaluated using quantitative real-time polymerase chain reaction (PCR) * = p＜0.05; VEGF = Vascular endothelial growth factor

VEGF levels peaked seven days after injury (Figure 3). Real-time PCR showed that VEGF expression was significantly higher in the CO_2_ group as compared to that in the control group after seven days (p < 0.05).

**Figure 4:**
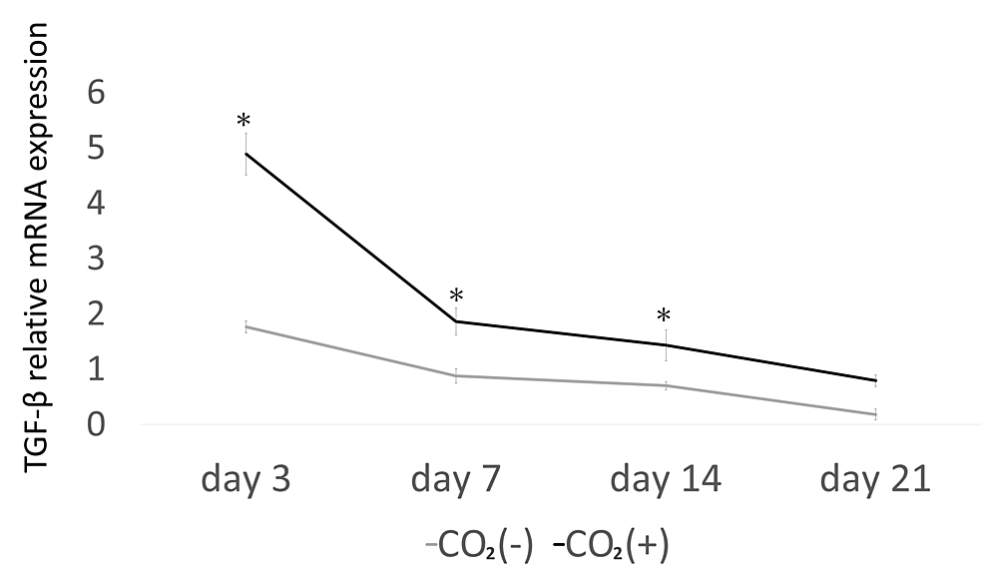
The mRNA expression of TGF-β. The mRNA expression was evaluated using quantitative real-time polymerase chain reaction (PCR) Vertical bars indicate standard deviation TGF-β = transforming growth factor-β; * = p ＜0.05

**Figure 5:**
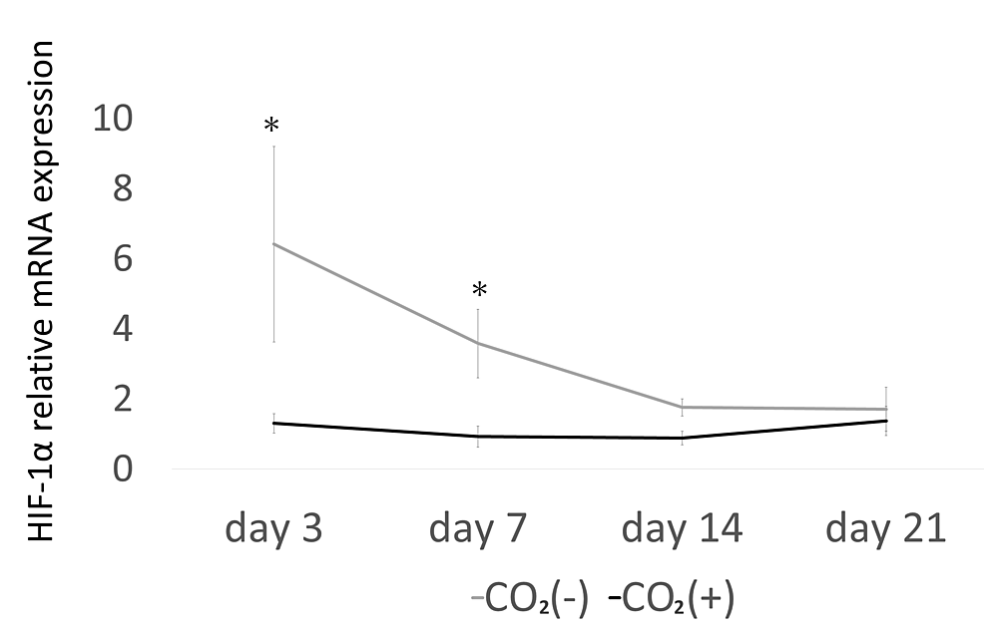
The mRNA expression of HIF-1α. The mRNA expression was evaluated using quantitative real-time polymerase chain reaction (PCR) HIF-1α = Hypoxia-inducible factor-1α; * = p ＜0.05.

**Figure 6:**
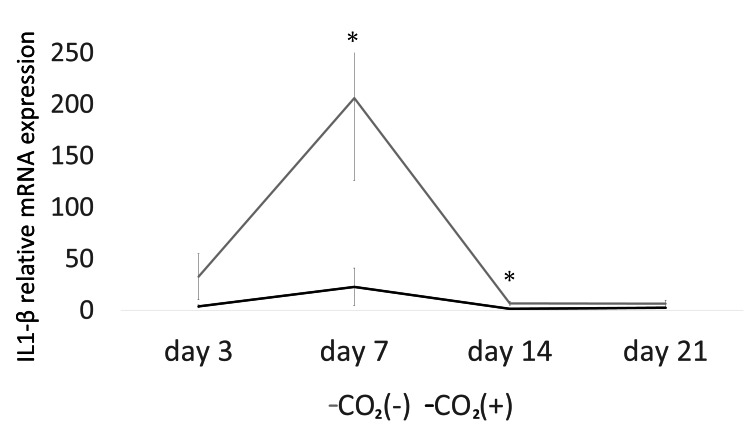
The mRNA expression of IL-1β. The mRNA expression was evaluated using quantitative real-time polymerase chain reaction (PCR). IL-1β = Interleukin-1β; * = p ＜0.05.

**Figure 7:**
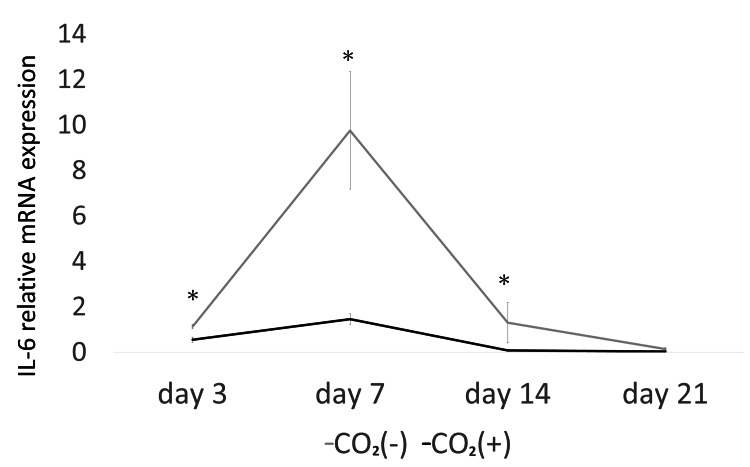
The mRNA expression of IL-6. The mRNA expression was evaluated using quantitative real-time polymerase chain reaction (PCR) IL-6 = Interleukin-6; * = p ＜0.05.

The expression of TGF-β (days 3, 7, and 14) was significantly higher in the CO_2_ group than that in the control group (p < 0.05; Figure 4). Furthermore, the expression of HIF-1α (days 3 and 7; Figure 5), IL-1β (days 7 and 14; Figure 6), and IL-6 (days 3, 7, and 14; Figure 7) was significantly lower in the CO_2_ group than that in the control group (p < 0.05). 

H&E staining

**Figure 8:**
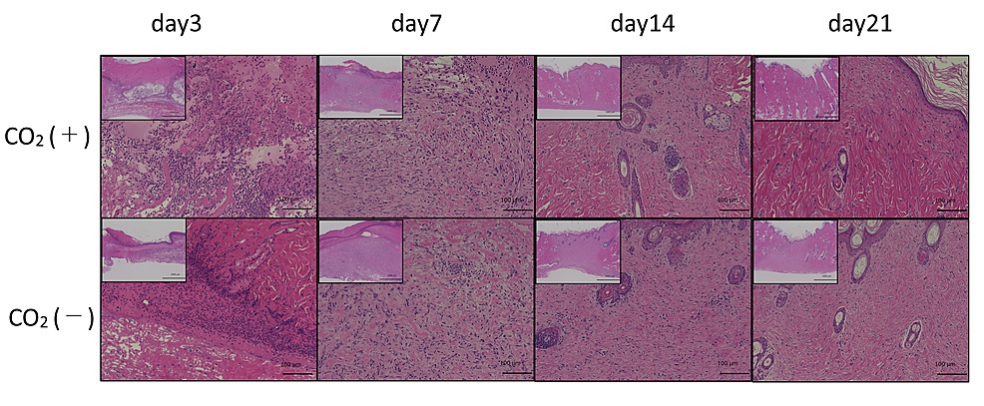
H&E histological observation of wounds in both groups on 3, 7, 14, and 21 days post-wounding Histological observation of wounds at ×40 (upper left corner, scale bar = 1000㎛) and ×200 (scale bar = 100㎛)

Figure 8 shows the H&E stained sections of wounds in the two groups on different days. On day 3, the site of the wound comprised inflammatory cells in both groups. On day 7, wounds in the control rats contained a small population of fibroblasts. However, rats from the CO_2_ group showed a higher abundance of proliferating fibroblasts. Moreover, fibroblasts were uniformly and densely arranged in the CO_2_ group as compared to the control group. On day 14, the fibroblast content gradually decreased in both groups. The rats from the control group showed remains of a scar. However, rats in the CO_2_ group showed better healing around the wound. Rats from the CO_2_ group showed uniformly arranged collagen fibers after 21 days. These rats only had a scar near the epithelium. In contrast, rats from the control group possessed a lower abundance of collagen fibers and a full scar.

Immunohistochemical analysis

**Figure 9:**
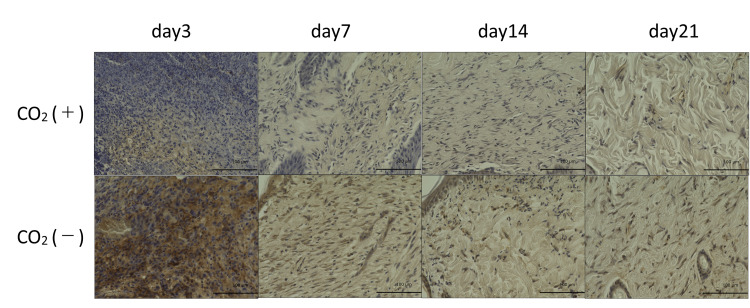
HIF-1α immunohistochemistry of wound sections of both groups on 3, 7, 14, and 21 days post-wounding HIF-1α = Hypoxia-inducible factor-1α Scale bar = 100㎛ and magnification ×400

**Figure 10:**
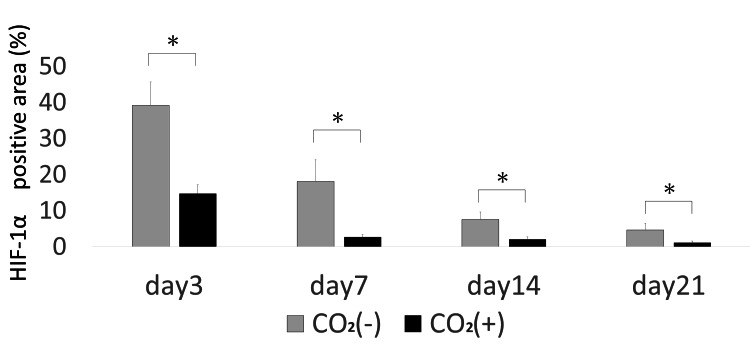
HIF-1α quantitative immunohistochemical analysis of the primary antibody-positive area ratio HIF-1α = Hypoxia-inducible factor-1α; * = p＜0.05

HIF-1α, VEGF, and TGF-β protein levels in the wounds were assessed using immunohistochemistry. HIF-1α-positive cells were more abundant in the control group across all the time points (Figures 9, 10).

**Figure 11:**
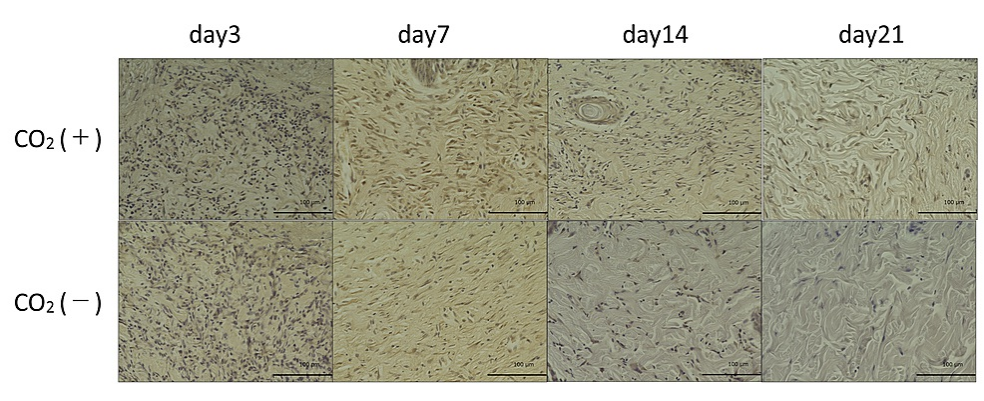
VEGF immunohistochemistry of wound sections of both groups on 3, 7, 14, and 21 days post-wounding VEGF = Vascular endothelial growth factor Scale bar = 100㎛ and magnification ×400

**Figure 12:**
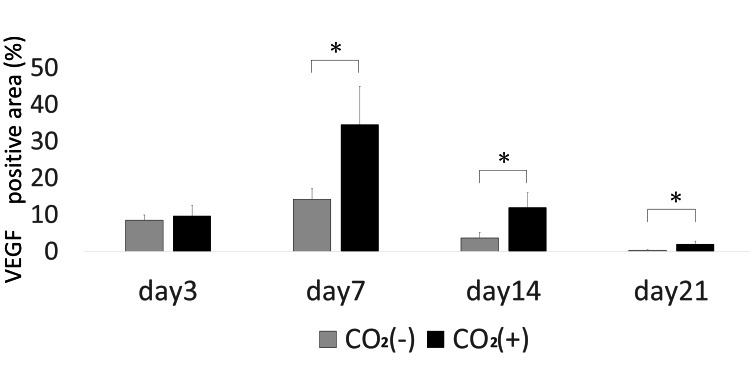
VEGF quantitative immunohistochemical analysis of the primary antibody-positive area ratio VEGF = Vascular endothelial growth factor; * = p＜0.05

The CO_2_ group showed higher VEGF expression as compared to that in the control group on days 7, 14, and 21 (Figures 11, 12).

**Figure 13:**
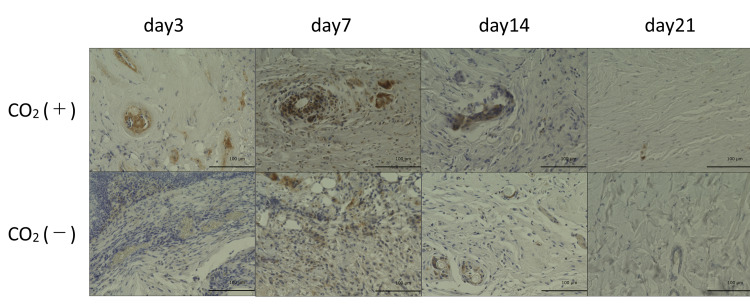
TGF-β immunohistochemistry of wound sections of both groups on 3, 7, 14, and 21 days post-wounding TGF-β = transforming growth factor-β Scale bar = 100㎛ and magnification ×400

**Figure 14:**
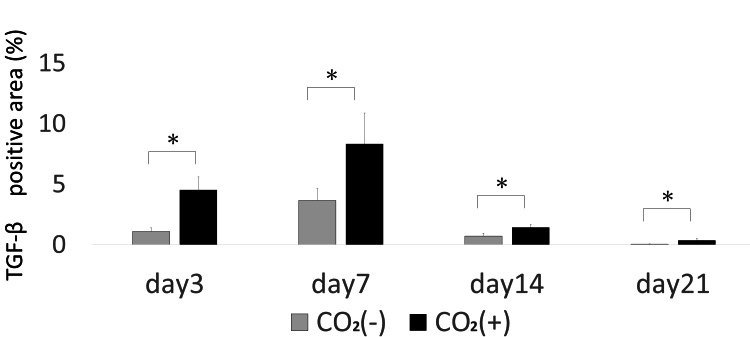
TGF-β quantitative immunohistochemical analysis of the primary antibody-positive area ratio TGF-β = transforming growth factor-β; * = p＜0.05

Moreover, the CO_2_ group showed higher levels of TGF-β as compared to that in the control group across all time points (Figures 13, 14). The data from immunohistochemistry were comparable to that obtained from real-time PCR. 

## Discussion

In this study, we have demonstrated that CO_2 _paste accelerated wound healing by upregulating VEGF and TGF-β and downregulating HIF-1α, IL-1β, and IL-6 in wounded rats.

CO_2_ therapy mechanistically involves the percutaneous absorption of CO_2_ that enhances vasodilation, blood flow, Bohr effect, and tissue O_2_ partial pressure. We have previously demonstrated that transcutaneous administration of CO_2_ results in an artificial Bohr effect by increasing tissue O_2_ pressure upon CO_2_ absorption [11]. Transcutaneous CO_2_ therapy has been used in humans and animal models to study its medical and cosmetic effects [12,13]. We have previously demonstrated that our transcutaneous CO_2_ system improves blood flow and increases angiogenesis [8,9]. Gaseous CO_2_ cannot be used in the head and neck region. In this study, we used a CO_2_ paste for wound healing that may be applied to the head and neck.

Wound healing is a complex process that involves multiple biological processes, such as inflammation, formation of granulation tissue, re-epithelialization, and matrix formation and remodeling [14]. Wound healing can be divided into three phases: inflammation, proliferation, and maturation [1]. Numerous cells, growth factors, and cytokines play an important role in wound healing. Thus, we investigated the roles of VEGF, TGF-β, HIF-1α, IL-1β, and IL-6.

HIF-1α is a protein that senses changes in environmental O_2_ and is involved in transcriptional regulation during hypoxia [15]. Hypoxia is an important prognostic determinant of wound repair and limits dermal wound healing [16]. Early-stage hypoxia induces wound healing, while prolonged hypoxia delays wound healing [17]. In this study, HIF-1α was upregulated in the control rats exposed to hypoxic stress. However, rats in the CO_2_ group failed to exhibit sustained hypoxia.

VEGF is produced by endothelial cells, fibroblasts, and macrophages and is important in wound healing. VEGF promotes the early events in angiogenesis, particularly endothelial cell migration and proliferation [14]. VEGF levels gradually increase between the early and middle stages of wound healing [2]. We have previously reported the CO_2_ therapy-induced increase in the expression of VEGF in rat fracture, skin flap, and muscle models [8,9,18]. In this study, VEGF levels significantly increased in the CO_2_ group on day 7. This indicates that the CO_2_ paste stimulates the expression of growth factors. Under hypoxia, VEGF is primarily regulated by HIF-1α [19]. VEGF is secreted at higher concentrations of O_2_ [20]. We observed a decrease in hypoxia and upregulation of VEGF. Thus, it may stimulate angiogenesis and wound healing.

TGF-β is important for angiogenesis and wound healing [1]. TGF-β is a multifunctional growth factor that exerts pleiotropic effects on wound healing by regulating cell proliferation, migration, differentiation, extracellular matrix production, and immune modulation [21]. TGF-β is produced by macrophages, fibroblasts, keratinocytes, platelets, and endothelial cells [14,22] and is important in inflammation, angiogenesis, re-epithelialization, fibroblast proliferation, collagen synthesis and deposition, and connective tissue regeneration [1,14,23]. Moreover, this growth factor functions in wound healing by facilitating fibroblast recruitment to the collagen matrix [24]. In this study, there was a significant increase in the mRNA levels of TGF-β in the rats of the CO_2_ group on days 3, 7, and 14.

Scarless wounds are characterized by a lack of inflammation, regeneration of dermal appendages, and orderly deposition of collagen [25]. Fibroblasts are cells that synthesize collagen and extracellular matrix that play a critical role in wound healing [26]. Gopal et al. reported enhanced wound healing in an open excision rat model and an increase in VEGF and TGF-β expression [26]. TGF-β is overexpressed in keloid tissues [27]. A gradual decrease in the expression of TGF-β indicates the suppression of excessive scar formation. Histopathologically, fibroblasts were arranged in a uniform and dense manner in the rats of the CO_2_ group on day 7. On day 14, the skin around the wound was replaced with collagen fibers that enabled wound shrinkage. The peak in the expression of TGF-β on day 7 in the CO_2_ group highlighted accelerated wound healing, implicating the CO_2_ paste in inducing normal healing. We observed a slight difference between our real-time PCR and immunostaining data for TGF-β. TGF-β mRNA levels peaked on day 3, while its protein levels peaked on day 7. This could be attributed to delayed protein synthesis.

An increase in inflammatory cytokines, such as IL-1β, prolongs inflammation and delays healing [28]. Hypoxia induces inflammation [29]. The levels of inflammatory cytokines (IL-6) and C-reactive protein increase under hypoxic stress [30]. Yu et al. showed the downregulation of IL-1β and upregulation of VEGF in a rat skin defect model with microcurrent dressing [2]. In this study, the expression of IL-6 (days 3, 7, and 14) and IL-1β (days 7 and 14) were lower in the rats of the CO_2_ group than that in the control rats. Therefore, CO_2_ paste might accelerate wound healing by regulating inflammatory cytokines and hypoxia.

However, this study has some limitations. First, this study involves an animal model and has not been conducted in humans. This is important since rat and human skin are different. Second, this study does not address the optimum conditions of CO_2_ treatment, such as application time and the number of days of treatment. Finally, the rats of the control group received only wound generation without any control paste. Therefore, the influences of treatment without a CO_2_ paste were not completely excluded. These limitations should be investigated in further studies.

## Conclusions

In summary, this study demonstrated that CO_2_ paste accelerated wound healing by upregulating VEGF and TGF-β and downregulating HIF-1α, IL-1β, and IL-6 in a rat model of skin defect. The application of CO_2_ paste accelerated normal wound healing by improving hypoxia and angiogenesis and reducing the expression of inflammatory cytokines. Further experiments, including human clinical studies, are needed to investigate the efficacy of CO_2_ paste on skin wounds. This will enable the development of novel strategies for patients with skin defects in the future.
